# Inflammatory Markers and Procalcitonin Predict the Outcome of Metastatic Non-Small-Cell-Lung-Cancer Patients Receiving PD-1/PD-L1 Immune-Checkpoint Blockade

**DOI:** 10.3389/fonc.2021.684110

**Published:** 2021-06-14

**Authors:** Valerio Nardone, Rocco Giannicola, Giovanna Bianco, Diana Giannarelli, Paolo Tini, Pierpaolo Pastina, Antonia Consuelo Falzea, Sebastiano Macheda, Michele Caraglia, Amalia Luce, Silvia Zappavigna, Luciano Mutti, Luigi Pirtoli, Antonio Giordano, Pierpaolo Correale

**Affiliations:** ^1^ Unit of Radiation Oncology, Ospedale del Mare, Naples, Italy; ^2^ Medical Oncology Unit, Grand Metropolitan Hospital “Bianchi-Melacrino-Morelli”, Reggio Calabria, Italy; ^3^ Biostatistical Unit, National Cancer Institute “Regina Elena”, IRCCS, Rome, Italy; ^4^ Section of Radiation Oncology, Medical School, University of Siena, Siena, Italy; ^5^ Unit of Intensive Care Medicine and Anesthesia, Grande Ospedale Metropolitano Bianchi Melacrino Morelli, Reggio Calabria, Italy; ^6^ Department of Precision Medicine, University of Campania “L. Vanvitelli”, Naples, Italy; ^7^ Laboratory of Precision and Molecular Oncology, Institute of Genetic Research, Biogem Scarl, Ariano Irpino, Italy; ^8^ Sbarro Institute for Cancer Research and Molecular Medicine and Center of Biotechnology, College of Science and Technology, Temple University, Philadelphia, PA, United States; ^9^ Department of Medical Biotechnology, University of Siena, Siena, Italy

**Keywords:** bacterial infections, immune-check point blockade, programmed cell death ligand-1, real-world salvage therapy, cell death receptor, prognostic factors of response, procalcitonin, inflammatory markers

## Abstract

Peripheral-immune-checkpoint blockade (P-ICB) with mAbs to PD-1 (nivolumab and pembrolizumab) or PD-L1 (atezolizumab, durvalumab, avelumab) alone or combination with chemotherapy represents a novel active treatment for mNSCLC patients. However, this therapy can be associated to immune-related adverse events (irAEs) and high cost. Therefore, finding reliable biomarkers of response and irAEs is strongly encouraged to accurately select patients who may potentially benefit from the immuno-oncological treatment. This is a retrospective multi-institutional analysis performed on ninety-five mNSCLC patients who received real-world salvage therapy with nivolumab or atezolizumab between December 2015 and April 2020. The outcome of these patients in term of PFS and OS was evaluated in comparison with different serum levels of C-reactive protein (CRP), Erythrocyte Sedimention Rate (ESR) and Procalcitonin (PCT) by performing Kaplan–Meier and Log-rank test and multivariate analysis. We found that high baseline levels of CRP, ESR, and PCT were strongly predictive of poor outcome (P <0.05) with the worse prognosis detected in those patients with a baseline levels of both ESR and PCT over the pre-established cut off (median OS recorded in patients with no marker over the cut off *vs*. those with just one marker over the cut off *vs*. those with both markers over the cut off: 40 ± 59 *vs*. 15.5 ± 5.5 *vs*. 5.5 ± 1.6 months, respectively; P <0.0001). Our results suggest the predictive value of systemic inflammation and suggest a potential role of PCT in predicting a poor outcome in mNSCLC receiving PD-1/PD-L1 blocking mAbs. This finding also suggests a potential role of subclinical bacterial infections in defining the response to PD-1/PD-L1 blocking mAbs that deserves further and more specific investigations.

## Introduction

Peripheral immune-check point blockade (P-ICB) with mAbs to the programmed cell death receptor-1 (PD-1) (nivolumab and pembrolizumab) and PD-Ligand (PD-L1) (atezolizumab, avelumab and durvalumab) alone or in combination with chemotherapy is a promising treatment option for metastatic non-small cell lung cancer (mNSCLC) (1, 2).

These innovative immune-oncological strategies are often very effective in the management of mNSCLC patients; however, they may be hampered by frequent more or less severe immuno-related adverse events (irAEs) and rising costs. Additionally, their fast-track evaluation in controlled trials has allowed their introduction in the clinical practice leaving a large amount of questions to be addressed (3, 4).

This treatment has substantial differences by other anticancer strategies like chemotherapy, radiotherapy and molecular target therapy that exert a direct cytotoxic/cytostatic effect on the tumor. On the other hand, PD-1-ICB does not act on the tumor cells but they rather rescue the antitumor activity of tumor infiltrating cytotoxic T cells (CTLs) mostly attenuated as consequence of PD-1 binding to PD-L1/2 in the tumor (1, 5, 6).

In this context, micro-environmental conditions related to chronic inflammation and/or relapsing infections might greatly affect the efficacy of these immune-effectors at several levels. These conditions may induce CTL exhaustion or promote the synthesis of pro-inflammatory cytokines like Interleukin (IL-6) and chemokines that, in turn, can have the following effects: i) to hamper the CTL-mediated response; ii) to enhance the production of immunosuppressive cell lineages (Treg, MSDC, M2 macrophages etc.) and iii) to promote the activation of multiple peripheral and central immune-checkpoints including those related to the hypoxic induced factor (HIF) and the adenosine receptor pathway (7–10).

This events might be of critical interest in mNSCLC patients who often present coexisting subclinical inflammatory and/or infectious conditions eventually related to the smoking habit, to chronic pulmonary obstructive disease symptoms and to the presence of relapsing infections within the low airways (11–13).

On these bases, we believe that the study of both inflammatory and infection markers in mNSCLC patients addressed to receive immune-checkpoint blocking mAbs could have a key role in understanding their effective interference in CTL activation and consequently on the outcome of these patients. At the present, the majority of the studies in the literature rely on the prognostic role of unspecific inflammatory markers such as white cell counts, NLR, CRP, ESR, LDH or more sophisticated techniques including tumor immune-profiling or microbiology studies (14–17).

Therefore, we have hypothesized that the presence of inflammatory markers mostly associated to bacterial infections of the low airways such as procalcitonin (PCT), might be easily correlated to the clinical outcome of mNSCLC patients subjected to immuno-oncological treatments. PCT, is a 116 amino acid peptide physiologically synthesized by thyroid parafollicular C cells that contribute to maintain the calcium homeostasis once converted to the calcitonin hormone and released in the blood stream (18–20). In the presence of a bacterial infection provoking a systemic inflammatory response, PCT synthesis may be induced in nearly all the involved tissues leading to a massive release of the peptide in the blood stream. On these bases, it is recommended as a reliable marker of typical bacterial infections, a feature not shared by other common inflammatory markers (18, 20). Systemic PCT production is triggered by bacterial toxins (endotoxin) and by pro-inflammatory cytokines such as tumor necrosis factor (TNF)-α, interleukin-1-β (IL-1β), and interleukin-6 (IL-6) as an immunological danger signal able to alert the host of a possible bacterial infection (18–24). Serum PCT is undetectable in healthy persons while it is greatly risen in patients bearing clinical or subclinical bacterial infections (20). On the other hand, PCT synthesis is not induced in most viral infections due to their ability to trigger the release of cytokines such as interferon (IFN)-γ that, in turn, inhibits the production of PTC inducers such as TNF-α (25–30).

At the same time, preliminary reports show that PCT in NSCLC could be correlated to survival, with a worse survival in patients with a PCT >0.1 ng/ml (31–33).

On the light of all these considerations, we carried out a retrospective study aimed to investigate whether the blood levels of PCT compared with conventional inflammatory markers such as CRP and ESR may predict the outcome of mNSCLC patients receiving PD-1/PD-L1 immune-checkpoint inhibitor mAbs.

## Materials and Methods

### Study Design and Patients

This work is part of a retrospective real-world evidence (RWE) multi-institutional database study including 95 chemo-refractory mNSCLC patients consecutively enrolled to receive salvage therapy with anti-PD-1 (nivolumab) or anti-PD-L1 (atezolizumab) mAb at the OU-RC, and ROU-SI between September 2015 and April 2020 with a median follow-up time of 28 months (34–36).

All the patients gave an informed consent for the anonymous use of their clinical data for the research aim. All procedures were undertaken in compliance with the ethical statements of the Helsinki Declaration (1964, amended most recently in 2008) of the World Medical Association and respect of their privacy. All patients received PD-1/PD-L1 blockade in *real world setting* as recommended by the international guidelines and regulatory agencies following the standard procedures of administration for each drug. All patients according to their specific disease received: nivolumab (intravenous infusion of 3 mg/kg every two weeks) (84 patients) or atezolizumab (intravenous infusion of 1,200 mg every three weeks) (33 patients) until disease progression or occurrence of severe adverse events. All patients were fit for treatment with no heart, kidney, and liver failure, no alterations in the blood cell counts and no clear sign of infection. All of the patients aimed to receive the treatment presented a good performance status ≤1 according to the Eastern Cooperative Oncology Group (ECOG). A complete physical examination report, histological sampling, hematologic, biochemical, immune-biological, radiological, and instrumental monitoring were available at baseline. Clinical history, physical examination, and record of adverse events were reported prior to each treatment cycle. A CT scan was performed at baseline and repeated every 3 months or in any case of suspected progressive disease (PD). CT scans were evaluated according to the immune Response Evaluation Criteria in Solid Tumors (iRECIST 1.1) (37).

All patients were monitored for blood cell counts, biochemistry, CRP, ESR, and PCT before each treatment course and were also monitored for their adrenal hormone profile, ACTH, TSH, thyroid hormones, anti-thyroid auto-antibodies (AAbs), extractable nuclear antigen antibodies (ENA), anti-nucleus antibodies (ANA), anti-smooth cells antibodies (ASMA), and c/p- anti-neutrophil cytoplasmic antibodies (ANCA) each month from the beginning of treatment as reported in previous study (38–40). Only pre-therapy (before the start of immunotherapy) parameters PCR, CRP and ESR were considered for the present analysis.

### Statistical Analysis

In order to perform a statistical correlation among continuous parameters and outcomes, we determined different cut-off for survival analysis (Kaplan–Meier analysis), on the overall population.

The PCT threshold chosen (0.1 ng/ml) was determined on the recent analysis from Kajikawa et al. (33), as it included only NSCLC patients and the same threshold was confirmed on multivariate analysis. For the other biomarkers (CRP, ESR), since a consensus in literature is lacking, we chose the median value as a cut-off (respectively CRP 1.6 mg/dl and ESR 40 mm/h).

Time to events was analyzed with the Kaplan–Meier method and statistics was performed by the log-rank test. Median survival and 95% confidence intervals were reported. Median follow-up was estimated with the reverse method. Hazard ratios (HRs) and their 95% confidence intervals were estimated through the Cox regression proportional model.

In the multivariate approach, a forward stepwise procedure was used and the enter and remove limit set to 0.05 and 0.10, respectively. Significant parameters at multivariate analysis were used to build a final model of survival, identifying different subgroups of patients. Chi-Square analysis was used to test the differences among the subgroup identified in terms of clinical variables.

Statistics were performed by the SPSS software 23.0 (International Business Machines Corp., New York, NY, USA).

## Results

### Patients’ Feature and Clinical Outcome

Our retrospective analysis was performed on a cohort of 95 patients with mNSCLC in our database who presented a parallel monitoring of serum CRP, ESR, and PCT values. In our series there were 77 males and 18 females who had been consecutively enrolled to receive salvage therapy with nivolumab (69 cases) or atezolizumab (26 cases) between November 2015 and April 2020 with a median follow of 28 months.

Patients’ demographic and clinical characteristics are summarized in **Table 1**.

**Table 1:** Clinical characteristics of the whole cohort of patients.

Characteristics	Percentage
**Sex**	
Males	77 (81.1%)
Females	18 (18.9%)
**Immunotherapy**	
Nivolumab	69 (72.6%)
Atezolizumab	26 (27.4%)
**Histology**	
Squamous	32 (33.7%)
Non-Squamous	63 (66.3%)
**Immune-related Adverse Events**	
Yes	32 (33.7%)
No	63 (66.3%)
**Age**	
<50 years	6 (4.2%)
50–65 years	38 (40%)
65–75 years	32 (33%)
>75 years	19 (20%)
**Expression of PD-L1 tumor expression, categorized**	
<1%	19 (20%)
1–50%	30 (31.5%)
>50%	15 (15.8%)
Missing	31 (32.7%)

All of the patients were fit for the immunological treatment and presenting an ECOG performance status in a range of 0–1; no clinical or radiological sign of active infection at baseline and no major impairment of heart, liver and kidney functions were recorded.

Mean age was 66 years ± 9.9 years, median age 67 years, range 32–85 years.

**Figure 1:**
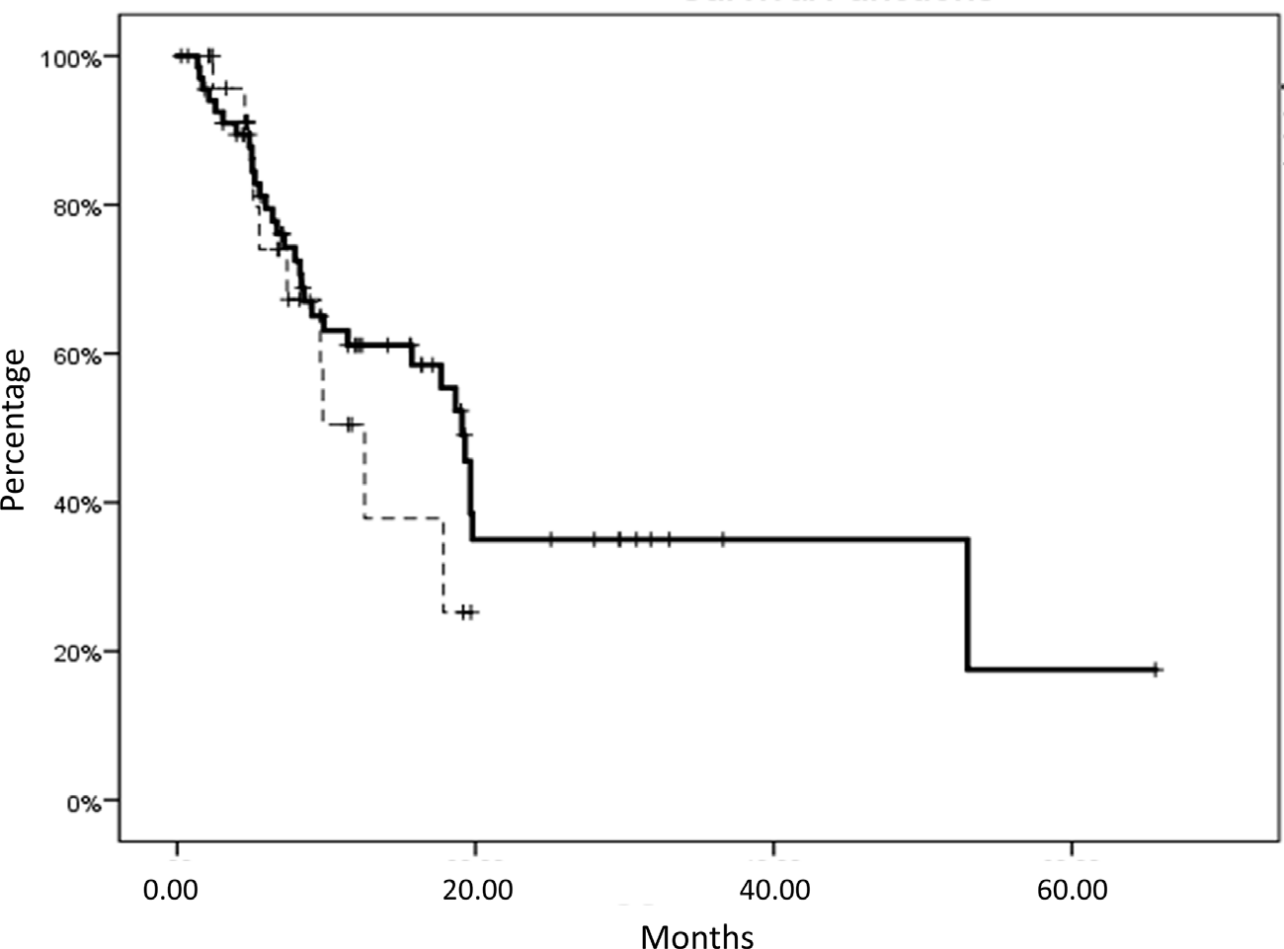
Overall survival (OS) of patients with metastatic non-small cell lung cancer (mNSCLC) subjected to nivolumab (solid line) or atezolizumab treatment (dashed line). No statistical differences in survival were correlated with the treatment (nivolumab *vs*. atezolizumab, p:0.378). Nivolumab subgroup median OS: 19 ± 0.8 months, mean OS: 27.9 ± 3.9 months, *versus* Atezolizumab subgroup median OS: 12.5 ± 2.1 months, mean OS: 12.1 ± 1.6 months.

On the overall, we recorded a median OS of 17.9 (95%CI; 11.0–24.6) months, with no statistical differences correlated with the treatment (nivolumab *vs*. atezolizumab, p: 0.378) (**Figure 1**). In this patients’ series we recorded irAEs in 33.7% of the patients (32/95 patients, grades 1–3) and no other adverse events or major organ failures unrelated to the malignant disease progression.

Haematological parameters mean value and standard deviations were as follow: PCT 0.21 ± 0.50 ng/ml, ESR 44.2 ± 31.4 mm/H, CRP 3.3 ± 5 mg/dl. All the evaluated parameters are reported in **Supplementary Materials**.

### Univariate Analysis of Survival

**Figure 2:**
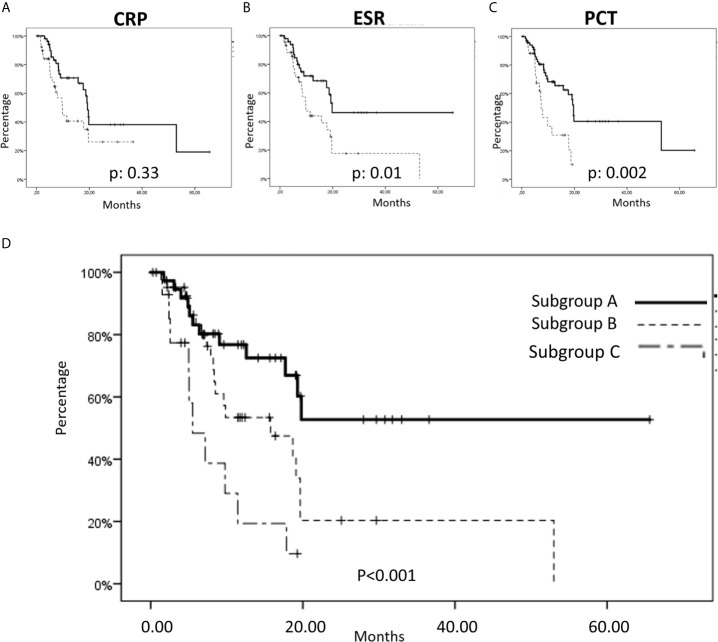
Overall survival (OS) considering inflammation status markers in metastatic non-small cell lung cancer (mNSCLC) patients. **(A–C)** In our series, we found a significant cut-off of the baseline expression of CRP, ESR and PCT that was strongly correlated to a worse outcome in terms of OS; **(A)** Specifically, patients with a CRP ≤16 showed a median OS of 19.3 ± 0.6 months, mean: 30.5 ± 4.7 months, versus a median OS of 9.7 ± 1.4 months, mean: 15.9 ± 27 months in patients with a CRP >16, with a p-value of 0.033; **(B)** Patients with ESR ≤40 showed a median OS of 19.8 ± 1.5 months, mean: 36.5 ± 5.4 months, versus a median OS of 9.8 ± 1.9 months, mean: 17.7 ± 3.5 months in patients with an ESR >40, with a p-value of 0.01; **(C)** Finally, patients with a PCT ≤0.1 showed a median OS of 19.6 ± 0.4 months, mean: 31.0 ± 4.3 months, versus a median OS of 7.3 ± 0.6 months, mean: 10.1 ± 1.4 months, in patients with a PCT >0.10, with a p-value of 0.002. **(D)** OS in tree subgroups of patients: Subgroup A (Baseline ESR values ≤40 mm/h and baseline PCT values ≤0.10 mg/L, were detected in 37/95 patients) showed a median OS not reached, mean 40 ± 5.9 months; Subgroup B (baseline ESR values >40 mm/h or baseline PCT values >0.10 mg/L were detected in 43/95 patients) showed a mean OS of 15.5 ± 5.5 months, mean: 20.1 ± 4.0 months; Sugbroup C (baseline ESR values >40 mm/h and baseline PCT values >0.10 mg/L were detected in 15/95 patients) showed a median OS of 5.5 ± 1.6 months, mean OS: 8.3 ± 1.7 months.

We carried out a statistical analysis aimed to compare the outcome of the patients presenting CRP, ESR and PCT baseline values below or over the respective pre-established cut-off value. The overall survival was obtained for each specific marker (**Figures 2A–C**). In details, patients presenting baseline values of CRP≤ *vs*. >16 mg/L showed a median OS of 19.3 ± 0.6 (mean: 30.5 ± 4.7) *vs*. 9.7 ± 1.4 (mean: 15.9 ± 2.7) months, respectively, with a p-value of 0.033. Additionally, patients presenting baseline values of ESR≤ *vs*. >40 mm/h showed a median OS of 19.8 ± 1.5 (mean: 36.5 ± 5.4) *vs*. 9.8 ± 1.9 (mean: 17.7 ± 3.5) months, respectively, with a p-value of 0.01. Finally, patients presenting baseline values of PCT ≤ 0.1 *vs*. >0.1 mg/L showed a median OS of 19.6 ± 0.4 (mean: 31.0 ± 4.3) *vs*. 7.3 ± 0.6 (mean: 10.1 ± 1.4) months, respectively, with a p-value of 0.002.

### Multivariate Analysis of Survival

Cox regression analysis of OS showed that only ESR (HR 2.11; 95% CI: 1.12–3.99; p = 0.02) and PCT (HR 2.64, 95% CI: 1.34–5.18; p = 0.005) were statistically significant. ESR and PCT were used to build a model of OS, dividing the cohort in three subgroups with the relative characteristics summarized in **Table 2**. Chi-Square analysis showed no significant difference among the three groups in terms of clinical variables (see **Table 2**). In the subgroup A were included patients (37 cases; 38.9%) with ESR and PCT baseline values equal to or below the respective cut-offs, who showed a prolonged OS (median OS not reached with a mean of 40 ± 5.9 months); in the subgroup B were included patients (43 cases; 45.3%) presenting just one of ESR or PCT baseline values over the respective cut off, who showed a median OS of 15.5 ± 5.5 (mean: 20.1 ± 4.0 months) months; finally, in the subgroup C were included patients (15 cases; 15.8%) with both ESR and PCT baseline values over the cut-off who showing the worst median OS of 5.5 ± 1.6 (mean: 8.3 ± 1.7) months. The differences among the three subgroups were statistically significant with a model p-value <0.001, HR 2.3, 95% CI 1.5–3.5 (**Figure 2D**).

**Table 2:** Clinical characteristics of the three cohort of patients.

Characteristics	Subgroup A	Subgroup B	Subgroup C
	n. 37 patients	n. 43 patients	n. 15 patients
**Sex** p-value: 0.785			
Males	29 (78.4%)	35 (81.4%)	13 (86.7%)
Females	8 (21.6%)	8 (18.6%)	2 (13.3%)
**Immunotherapy** p-value: 0.818			
Nivolumab	28 (75.7%)	31 (72.1%)	10 (66.7%)
Atezolizumab	9 (24.3%)	12 (27.9%)	5 (33.3%)
**Histology** p-value: 0.973			
Squamous	12 (32.4%)	15 (34.9%)	5 (33.3%)
Non-squamous	25 (67.6%)	28 (65.15)	10 (66.7%)
**IrAEs** p-value: 0.465			
Yes	13 (35.1%)	16 (37.2%)	3 (20%)
No	24 (64.9%)	27 (62.8%)	12 (80%)
**Age** p-value: 0.156			
<50 years	1 (2.7%)	2 (4.7%)	2 (13.3%)
50–65 years	15 (40.5%)	17 (39.6%)	6 (40%)
65–75 years	13 (35.1%)	15 (34.8%)	6 (40%)
>75 years	8 (21.7%)	9 (20.9%)	1 (6.7%)
**Expression of PD-L1 expression, categorized** p-value: 0.818			
<1%	6 (16.2%)	9 (20.9%)	4 (26.6%)
1–50%	11 (29.8%)	15 (34.9%)	4 (26.7%)
>50%	8 (21.6%)	6 (13.9%)	1 (6.7%)
Missing	12 (32.4%)	13 (30.3%)	6 (40%)

Subgroup A included 37 patients with baseline ESR values ≤40 mm/h and baseline PCT values ≤0.10 ng/L. Subgroup B included 43 patients’ baseline ESR values >40 mm/h or baseline PCT values >0.10 ng/L. Subgroup C included 15 patients with baseline ESR values >40 mm/h and baseline PCT values >0.10 ng/L. Chi-Square analysis showed no differences in clinical variables among the three groups.

## Discussion

Our retrospective multi-institutional analysis performed in real world setting and aimed to evaluate the effects of either inflammatory and/or infection markers in mNSCLC receiving PD-1/PD-L1 blocking mAbs, showed a direct correlation of CRP, ESR and PCT baseline values with a poor outcome in terms of survival. The PCT threshold chosen was determined on the recent analysis from Kajikawa et al. (33), as it included only NSCLC patients and the same threshold was confirmed on multivariate analysis. Conversely, other analyses included generally patients with lung cancer (both SCLC and NSCLC) and concluded that PCT is elevated in patients with lung cancer with neuroendocrine component or with metastases (31, 32). For the other biomarkers (CRP and ESR), since a consensus in literature is lacking, we chose the median value as a cut-off. The arbitrary choice of the cut-off must be recognized as a limitation of our retrospective study.

These results, however, are in line with our previous results and with what reported by several authors concerning the negative influence of inflammation on the outcome of patients subjected to palliative chemotherapy, radiotherapy and/or immunomodulating treatments for mNSCLC. On the basis of these data, it can be hypothesized that a coexisting chronic inflammation in mNSCLC patients may affect the immune-balance between the immune-system and cancer growth affecting the anti-tumor activity of T cells and promoting the mechanisms of cancer immune-escape (39–41).

At the same time, additional analysis on the correlation between inflammation parameters and outcomes in the specific setting of immunotherapy is needed, in order to investigate whether these parameters are simply prognostic biomarkers (i.e.: they are correlated to the prognosis of lung cancer patients independently from the choice of the systemic therapies) or predictive biomarkers of response to immunotherapy.

It has already been shown that chronic inflammation is commonly associated to the production of cytokines/chemokines that can hamper the efficient CTL response; to the rise of immune-suppressive cell lineages (including T_reg_s and MDSCs) and to the triggering of the activation of multiple immune-checkpoints and immune-suppressive adenosine receptors (42, 43).

The presence of inflammatory processes in mNSCLC patients may be related to the presence of malignancy itself, to concomitant smoke-associated bronchopulmonary chronic disease and to the presence of relapsing bacterial infections related to incomplete integrity the airways (44–49).

In this light, there are several evidences on the impairment of the existing balance between immune cell-mediated destruction and growth of cancer cells during a long-lasting inflammatory status in cancer patients. This can indeed result in an accelerated progression of the disease and a worse prognosis. In this context, our results highlighting the ability of PCT, an inflammatory marker associated to gram-positive bacterial infection, to predict the outcome of mNSCLC patients receiving immunotherapy, are in line with the results of other authors who recently showed that high baseline levels of PCT are predictive of a poor prognosis in mNSCLC (31, 33, 50).

It is noteworthy to underline that in the setting of mNSCLC an increase of infection biomarkers, such as PCT, CRP and ESR, cannot be considered very specific for active infections, as these biomarkers may be related to the malignancy itself or to concomitant smoke-associated bronchopulmonary chronic disease (32), although PCT can be considered more specific and aid in the differential diagnosis between infectious fever and tumor fever (50, 51).

However, in our series we observed, in a multivariate analysis, that patients presenting at the same time ESR and PCT baseline values over the cut-off showed the worse outcome (mean OS = 8.3 ± 1.7 months) compared with the outcome recorded in patients with just one of the two marker values over the cut-off (mean OS = 20.1 ± 4.0 months) or in patients with both marker values below the cut-off (mean OS = 40 ± 5.9 months). This finding suggests that both inflammation and infection have an additive and independent detrimental effect on the outcome and on the treatment response to the immuno-oncological treatment with anti PD-1 and PD-L1 blocking mAbs.

PCT is considered a serum biomarker able to distinguish bacterial infection from other causes of infection or inflammation. This could be of particular interest in patients with infections of the lower respiratory tract where PCT may help in resolving diagnostic uncertainty and guiding to antibiotic therapy; in details, antibiotics’ discontinuation in patients with pneumonia, is commonly recommended on defined PCT thresholds (below 0.25 or 0.5 ng/ml or decrease by ≥80% from peak if initial value is >5 ng/ml) in combination with clinical judgment (19, 52, 53). It is conceivable that this recommendation might be still valid in patients bearing a malignant disease and need to be investigated in appropriate trials.

It should be stated that not all bacterial infections cause similar PCT increase. The most common infections by typical bacteria such as *Streptococcus pneumonia* and* Haemophilus influentia*, are commonly associate to higher PCT levels (median blood values of 2.5 ng/ml) compared with atypical bacteria (like *Mycoplasma*, *Legionella*, *Chlamydia*, *Mycobacterium tubercolosis*, etc.), or other eukaryotic parasites (alike *Candida* and *Pneumocystis species*), (median blood values of 0.20 ng/ml) and viruses (median blood values of 0.09 ng/ml) (19, 52). On the other hand, systemic inflammation not associated with pathogens, including cancer (with the exception of neuroendocrine malignancies), shock, injuries and chronic kidney disease and more severe autoimmune diseases have a limited effect on PCT levels.

However, unspecific PCT rises have been sporadically reported upon treatment with immunomodulatory treatments such as T-cell antibodies, alemtuzumab, IL-2, and granulocyte transfusions even though in our setting we were unable to show any significant increase in CRP, ESR and PCT related to the use of mAbs to PD-1 or PD-L1 (54–56).

Serum PCT blood levels commonly rise within few hours after the microbiological insult and roughly correlate with the severity of infection declining at a predictable fast rate with complete resolution. However, it should be taken in consideration that PCT production continues maintaining a plateau level when the infection/inflammatory stimulus is not completely solved (57–59). On these bases of the results derived from multiple observational studies, it has been hypothesized that PCT levels over the thresholds, reflect the existence of an active bacterial infection even though other clinical signs and symptoms are missing. Additionally, there is also evidence that the speed of rising in PCT serum levels correlates with the severity of the infection, a fact which allow the physicians to rely on this marker to monitor the systemic evolution of the ongoing infectious disease and the efficacy of the antibiotic therapy in elderly and cancer patients where the morbidity from bacterial infections is unpredictable and often lethal (60–64).

As an additional consideration, it is important to take in consideration the in mNSCLC patients receiving immuno-oncological treatments the role of the specific broncho-pulmonary microbiota that in analogy with what reported for the intestinal resident bacteria, may affect the immunological response of the host to the tumor and consequently the efficacy of immune-checkpoint inhibitors. In this context, it has been recently shown that the unspecific use of antibiotics prior to PD-1 blockade is correlated with a poor response to the treatment and it is commonly not advised, although some concerns of bias related to the selection of patients (65–71). In this regard, the use of infectious biomarkers such as PCT could help to properly select patients with active infections requiring antibiotics and to spare the unspecific use of these drugs avoiding their detrimental effects on specific microbiota.

Also, we did not find any correlation between the inflammation biomarkers and the development of irAEs. At this regard, we can speculate that basal parameters cannot predict the immune-related side effects, unfortunately, as immunotherapy has not started yet. We believe that the dynamic evaluation of these biomarkers during the course of immunotherapy (i.e.: in different time points, before each cycle of immunotherapy) could help in this prediction. A proper investigation of this phenomenon is needed in the next future with a dedicated prospective trial.

Our work recognizes the limitation of a monocentric retrospective study that deserves validation in external datasets and/or prospective validation. Presently, external validation was not possible, as PCT is not included in the routinary clinical management of mNSCLC.

In the present study we describe the detrimental effect of systemic inflammation and infection in mNSCLC receiving PD-1/PD-L1 blockade immunotherapy and suggest a perspective investigation of CRP, ESR and PCT as potential biomarker of response to the immuno-oncological treatment. The results of this study also open a new research scenario potentially played by bacterial influence and eventually on the preventive use of antibiotics in patients with high baseline levels of ESR and PCT, aimed to receive immune-checkpoint blockade.

## Data Availability Statement

The raw data supporting the conclusions of this article will be made available by the authors, without undue reservation.

## Ethics Statement

Ethical review and approval was not required for the study on human participants in accordance with the local legislation and institutional requirements. The patients/participants provided their written informed consent to participate in this study.

## Author Contributions

VN, DG, MC, and PC conceived and designed the study and wrote the manuscript. RG, GB, PT, PP, AF, SM, and PC acquired the clinical data. DG performed statistical analysis. VN, LM, LP, AG and PC followed the patients, including planning clinical visits, blood sample collection and follow-up. AL, MC, PC and SZ revised the paper. All authors contributed to the article and approved the submitted version.

## Conflict of Interest

The authors declare that the research was conducted in the absence of any commercial or financial relationships that could be construed as a potential conflict of interest.
