# Supplementary material for: Inflammatory Markers and Procalcitonin Predict the Outcome of Metastatic Non-Small-Cell-Lung-Cancer Patients Receiving PD-1/PD-L1 Immune-Checkpoint Blockade
Source: Front Oncol. 2021 Jun 14;11:684110. doi: 10.3389/fonc.2021.684110 (PMC8236817; doi:10.3389/fonc.2021.684110)
Supplement: Supplementary file 1 [file Table_1.docx]

Supplementary Materials

Table 1: Each patient’s individual CRP, PCT and ESR values are provided in the table

| Number | PCT | ESR | LDH | RCP |
| --- | --- | --- | --- | --- |
| 1 | ,12 | 75,00 | 470,00 | 3,70 |
| 2 | ,31 | 22,00 | 177,00 | ,70 |
| 3 | ,07 | 32,00 | 420,00 | ,30 |
| 4 | ,03 | 11,00 | 446,00 | ,40 |
| 5 | ,04 | 53,00 | 400,00 | ,30 |
| 6 | ,05 | 49,00 | 225,00 | 2,00 |
| 7 | ,03 | 57,00 | 400,00 | 4,20 |
| 8 | ,04 | 71,00 | 219,00 | 2,10 |
| 9 | ,03 | 34,00 | 229,00 | ,40 |
| 10 | ,09 | 6,00 | 479,00 | ,30 |
| 11 | ,08 | 56,00 | 336,00 | 1,50 |
| 12 | ,09 | 98,00 | 412,00 | 8,50 |
| 13 | ,11 | 43,00 | 1868,00 | 11,80 |
| 14 | ,10 | 40,00 | 443,00 | 1,80 |
| 15 | ,08 | 51,00 | 390,00 | ,30 |
| 16 | 1,60 | 6,00 | 252,00 | 2,40 |
| 17 | ,07 | 60,00 | 191,00 | ,90 |
| 18 | ,09 | 43,00 | 233,00 | 7,00 |
| 19 | ,10 | 79,00 | 1550,00 | 9,80 |
| 20 | ,08 | 22,00 | 457,00 | ,30 |
| 21 | ,06 | 120,00 | 193,00 | 5,20 |
| 22 | ,03 | 21,00 | 199,00 | ,30 |
| 23 | ,03 | 12,00 | 388,00 | ,20 |
| 24 | ,78 | 12,00 | 707,00 | ,30 |
| 25 | ,20 | 25,00 | 827,00 | 1,80 |
| 26 | ,12 | 23,00 | 711,00 | 2,70 |
| 27 | ,06 | 64,00 | 178,00 | 1,60 |
| 28 | ,17 | 36,00 | 191,00 | ,30 |
| 29 | ,08 | 28,00 | 170,00 | 1,90 |
| 30 | ,10 | 53,00 | 209,00 | 2,50 |
| 31 | ,13 | 115,00 | 506,00 | 14,60 |
| 32 | ,08 | 33,00 | 436,00 | 5,90 |
| 33 | 2,22 | 66,00 | 418,00 | 9,20 |
| 34 | ,14 | 120,00 | 283,00 | 7,50 |
| 35 | ,08 | 91,00 | 390,00 | ,80 |
| 36 | ,11 | 44,00 | 149,00 | 12,50 |
| 37 | ,09 | 42,00 | 850,00 | 2,30 |
| 38 | ,10 | 54,00 | 217,00 | 2,40 |
| 39 | ,13 | 25,00 | 483,00 | 2,60 |
| 40 | ,08 | 17,00 | 195,00 | ,30 |
| 41 | ,02 | 19,00 | 190,00 | ,70 |
| 42 | ,21 | 77,00 | 151,00 | 5,70 |
| 43 | ,04 | 90,00 | 304,00 | 8,60 |
| 44 | ,05 | 14,00 | 191,00 | ,30 |
| 45 | ,04 | 6,00 | 176,00 | 5,30 |
| 46 | 1,51 | 120,00 | 432,00 | 3,80 |
| 47 | ,00 | 23,00 | 590,00 | ,40 |
| 48 | ,09 | 38,00 | 806,00 | 2,70 |
| 49 | ,22 | 33,00 | 471,00 | 2,10 |
| 50 | ,11 | 42,00 | 550,00 | 2,60 |
| 51 | ,07 | 34,00 | 212,00 | 1,20 |
| 52 | ,07 | 11,00 | 411,00 | ,30 |
| 53 | ,04 | 56,00 | 188,00 | 2,40 |
| 54 | ,08 | 13,00 | 566,00 | 1,90 |
| 55 | 3,24 | 120,00 | 394,00 | 23,60 |
| 56 | ,13 | 120,00 | 235,00 | 4,90 |
| 57 | ,10 | 58,00 | 510,00 | 4,50 |
| 58 | ,03 | 53,00 | 273,00 | 1,80 |
| 59 | ,15 | 23,00 | 378,00 | ,40 |
| 60 | ,08 | 52,00 | 478,00 | ,90 |
| 61 | ,05 | 78,00 | 211,00 | 5,10 |
| 62 | ,10 | 15,00 | 414,00 | ,30 |
| 63 | ,10 | 43,00 | 343,00 | ,30 |
| 64 | ,10 | 54,00 | 643,00 | 8,20 |
| 65 | ,03 | 7,00 | 434,00 | ,30 |
| 66 | ,10 | 20,00 | 593,00 | ,30 |
| 67 | ,08 | 34,00 | 190,00 | ,40 |
| 68 | ,03 | 10,00 | 361,00 | 2,50 |
| 69 | 2,49 | 70,00 | 517,00 | 31,60 |
| 70 | ,06 | 52,00 | 579,00 | ,60 |
| 71 | ,18 | 13,00 | 503,00 | ,40 |
| 72 | ,04 | 6,00 | 380,00 | ,30 |
| 73 | ,03 | 82,00 | 465,00 | 1,60 |
| 74 | ,04 | 21,00 | 158,00 | ,80 |
| 75 | ,04 | 10,00 | 206,00 | ,30 |
| 76 | ,03 | 36,00 | 236,00 | ,40 |
| 77 | ,10 | 46,00 | 450,00 | ,30 |
| 78 | ,10 | 31,00 | 400,00 | 5,50 |
| 79 | ,05 | 7,00 | 358,00 | 1,00 |
| 80 | ,26 | 55,00 | 375,00 | 2,10 |
| 81 | ,06 | 12,00 | 156,00 | ,30 |
| 82 | ,65 | 104,00 | 734,00 | 17,80 |
| 83 | ,10 | 8,00 | 154,00 | ,50 |
| 84 | ,03 | 25,00 | 357,00 | ,40 |
| 85 | ,05 | 7,00 | 158,00 | 1,10 |
| 86 | ,09 | 9,00 | 293,00 | ,30 |
| 87 | ,08 | 89,00 | 668,00 | 2,60 |
| 88 | ,14 | 39,00 | 226,00 | 12,10 |
| 89 | ,07 | 55,00 | 465,00 | ,30 |
| 90 | ,12 | 52,00 | 306,00 | ,60 |
| 91 | ,09 | 7,00 | 1206,00 | ,30 |
| 92 | ,09 | 90,00 | 349,00 | 2,10 |
| 93 | ,08 | 10,00 | 400,00 | 5,90 |
| 94 | ,07 | 58,00 | 570,00 | ,40 |
| 95 | ,11 | 40,00 | 249,00 | 1,20 |
